# Potency of some economic variables affecting environmental quality in Nigeria

**DOI:** 10.1038/s41598-023-34968-1

**Published:** 2023-06-28

**Authors:** T. O. Ogunbode, J. P. Jazat, J. A. Akande

**Affiliations:** Environmental Management and Crop Production Unit, College of Agriculture, Engineering and Science, Bowen University, Iwo, Nigeria

**Keywords:** Environmental sciences, Environmental social sciences

## Abstract

Environmental quality (EQ) is germane to achieving sustainable living on earth. To evaluate a related stimulus of EQ on area basis, a comparative analyses of economic factors that influence pollution in Iwo and Ibadan metropolis was carried out. Data for the study was generated through the administration of 700 structured questionnaires in total at both locations, out of which 165 and 473 were retrieved from Iwo and Ibadan respectively. The distribution of respondents in form of male gender, married status, tertiary education and household size of not more than 5 were 51.5%, 78.2%, 24.9% and 46.1% respectively for Iwo, while it was 38.5%, 81.0%, 28.6% and 48.8% in the same order for Ibadan. Economic factors analyzed were (1) Income (2) living standard indexed by the form of accommodation occupied (3) waste/noise management regimen (4) energy utilization (5) orthodox versus green economy adoption and (6) waste sorting capacity. Kaiser–Meyer–Olkin sampling adequacy and Bartlett’s test of sphericity admitted the data as factorable at *p* < 0.005. Results showed that three of the economic variables significantly explained the pollution status in Iwo and Ibadan. The variables in Iwo explained 59.3% of the factors and these are waste/noise management strategies (22.5%), living standard (18.7%) and green economy adoption (18.3%). 60.2% of economic impacts on pollution in Ibadan were explained by living standard (24.4%), green economy adoption (18.8%) and waste/noise management strategies (17.0%). Only two of the variables namely living standard, and green economy adoption were common to the two study locations, although, their importance and ordering varied. While waste and noise management were most significant in Iwo, the same variable had the least effect in Ibadan. Green economy adoption was least in Iwo but most significant in Ibadan. Thus, the economic factors influencing pollution in Iwo and Ibadan metropolis, though similar, may not be given a generalized weighting. In other words, analyses of pollution-related matters from the economic viewpoint should be location specific.

## Introduction

An environment that is pollution-free forms one of the global desires targeted to be actualized by 2030 through United Nations Sustainable Development Goals (SDGs). European Environmental Agency^1^ defined environmental quality(EQ) as “a term which refers to varied characteristics such as air and water purity or pollution, noise, access to open space, and the visual effects of buildings, and the potential effects which such characteristics may have on physical and mental health”. According to^2,3^, the benefits of sustainable environmental quality is not far-fetched- it is the driver of economic growth which manifests in productive economy, food security, industrial development and growth, life security, sound financial development, good governance, full employment, crime-free society to mention but a few. No wonder that the United Nations has set before it indomitable environmental quality to achieve sustainability of life through Goal 11. (Sustainable Cities and Communities). One of the targets of Goal 11 stated that ***there should be reduction in the adverse per capital environmental impact of cities, including by paying attention to air quality and municipal and other waste management by 2030***^4^.

Several attempts have been made to ensure that sound EQ in human society such as general cleanliness, tree planting, landscaping, isolating industrial areas from residential areas, among others. However, all these efforts have not yielded the expected results of sound EQ for human sustenance, but rather still seem to compound the challenge. For instance, the segregation of industrial areas from residential areas has not removed environmental pollution because such by-products eventually still found their ways into the global space whether residential or not. Carbon emission is not also confined to only where carbon was dislodged like through deforestation and so on. Environment is always under threat by human activities. Ogunbode et al.^5–7^ had respectively investigated the impact of oil palm mill on some natural resources and human trading activities on urban environment.

The introduction of contaminants into the environment is mostly anthropogenic. Man engages in several activities which exert negative consequences on the environment. Such activities include agriculture, wood logging, industrial establishments, all of which were necessities for man to sustain his livelihood. Apart from these activities, some other actions towards his environment also pose threats to the natural environment which could be addressed for sustainable EQ. For instance, activities such as indiscriminate dump of refuse, agro-chemical applications on farms, urbanization, non-adherence to urban planning directives, inadequate knowledge about the significance of environment to human living among others, most of which are commonly found in the developing countries^8^. In another instance, Wang et al.^9^ and Coccia^10^ lamented on the trend noticed in the degraded quality of the natural resources and thus recommended intensified on public enlightenment to raise increased consciousness in environmental protection and management. Coccia^10^ reported that income disparity in their respective study areas made significant improvements in the control of air pollution. In addition, the impact of religious activities on air and noise pollution had been reported^11,12^ while^13,14^ investigated the impact of change in household environment condition on morbidity in India. All these poor attitude and inactions against the natural environment have partly resulted in dwindling human health, death, and economic downturn in most of these countries. The contemporary clashes between the Fulani herders and the crop farmers in Nigeria was partly attributed to environmental degradation—shortage of fodders to feed animals contributed to the southward movement of the herders with their animals, the process that has led to the encroachment into crop farms. Apart from this, the scenario of the prevailing global climate change, otherwise called global warming was also attributed to the reckless manner by which man has been handling the natural environment^15^. Albeit, trees are removed without attempt to replant it, thus given room for carbon displacement into the atmosphere. Despite the establishment of separate Ministry to cater for the environment in Nigeria, Unigwe and Egbueri^16^ lamented the way environmental laws and rules are flouted without any commensurate discipline. Refuse were dumped anywhere on the streets, water courses and public places in most urban centres without regards to the potential implications on the environment. Business units are lackadaisically located anywhere in urban centres and suburbs without following the laid down rules. Sporadic rise in the population in most the developing countries has also intensified the challenge of continuous falling in the quality of the natural environment. Thus, environmental degradation could result in epidemics and human deaths if not checkmated. It should be noted that all these challenges vary in magnitude and scales in space and needs to treated in tandem to the scales.

Despite the outcries of the global community at regional and local levels on the prevalent degradation of the environment and the need to put it under control, most government still lack the political will to act on the challenge. Ogunbode and Asifat^17^ and Coccia^18^ decried that such attitude from political class is often attributed to conflicts with other pressing needs and/or cost implication of executing such measures. Unigwe and Egbueri^16^ bemoaned the poor attitude of Nigerian government to comply with court rulings on matters relating to environment brought before the court and so considered strengthening legal institutions and keeping to international treaties on environment sanity which Nigeria was part of as ways to enjoy the benefits of EQ. Coccia^19^ had also challenged the government in Nigeria on the consequences of air pollution in his investigation which has impacted on life expectancy in the country. Nwani, therefore posited that the government should implement measures that will improve air quality and public enlightenment that will checkmate air pollution in the country.

Different publications on the EQ subject matter are available in print and in digital versions with discussion from diverse perspectives such as causes, types, effects and control of EP^20–22^. Despite this, investigations are still scanty on how economic variables contribute to EQ. This is true of most developing nations like Nigeria. It should be noted that environmental pollution has been observed to have correlations with certain economic variables such as income levels, industrial production, gross domestic product, to mention but a few. It is expected that solutions to EP in low income environment should differ from that of high income environment. Today in Nigeria, similar measures are executed to checkmate environmental pollution, which probably is the reason why every effort of the management is yet to yield the expected positive results. It is unfortunate that with all efforts of the stakeholders in pollution management, the problem keeps persisting in the country^23^. Thus, this study is desirable to evaluate the contributions of economic variables to EQ. A comparative investigation as this is expected to expand the frontiers of knowledge on the subject matter, especially on thoughts renewal on tackling EP based on variations in the economic variables in the study areas. The objectives are; (1) identification of the economic variables that influence environmental pollution in the study areas and (2) determination those economic variables that are significant to explaining environmental pollution, and; (3) ordering of the significant variables in the order of their contribution to pollution. It is expected that the outcome of this research will enable relevant management to re-consider their strategies on the control of pollution for improved environment in the study area.

## Literature review and the theoretical basis

Several factors are available in literatures with regards to the determinants of environmental quality. Guo et al.^22^, Ekundayo and Nwachukwu^24^, Manisalidise et al.^25^, examined the consequences of oil palm mils set-ups on environmental resources such as soil, water, plants, and microorganisms. However, the health implication of environmental pollution needs to be examined because a quality environment is a driver of sound human health and EG. Some of the works on the det4erminants of environmental quality include^26^. Environmental quality is an investment that all and sundry should be involved in because it is a strong driver of economic growth and prosperity. Thus^27^, revealed that citizens’ willingness to pay (WTP) for environmental quality improvement is a right step in the right direction if EG will be realized and sustained. Wang et al.^27^ stated that environmental protection is a necessity to ensure sound public health and sustainable economic growth.

One of the quests of any human endeavour is to achieve economic growth. Economic growth (EG), when detached from any defined political space results in stagnation or worsen economy. An experience of EG implied economic prosperity, self-sustained, sustainable employment rate, financial buoyancy and liberation in the committee of nations^28^. EG further implies industrial development, self-reliance and less dependent of foreign economies, enhanced food security, educational development, viable productive economy, among others. Despite the growth accomplished, the implied misfortunes in that same economy are not unexpected, though the characterized growth in such economy should expectedly incorporate measures that will subdue or mitigate an implied negative impacts in that economy.

According to Islam^28^, EG albeit leads to degraded ecosystem and also brings about environmental pollution and degradation. In his investigation^28^, substantiated this fact when it was discovered that energy consumption and urbanization were significant factors in explaining environmental pollution (EP) in the South Asia with the aid of environmental Kuznets curve (EKC). It was further authenticated that both urbanization and energy consumption were strong influencers of EP while industrial value added was neutral, thus, it was suggested that rural–urban development should be enhanced to subdue EP. In a similar investigation^29^, showed that the impact of economic growth target (EGT) on EP is quantifiable. It was observed that EGT was a significant factor that aggravates EP in the region. It was revealed that EGT aggravates EP through investment surge, technological innovation and resource allocation. As a result of several impacts of EP in the global economy at regional and local scale, many nations have become integrated for the need of sustainable development and circular economy (CE) development.

Nie et al.^30^, in another perspective highlighted the impact of ethnic fractionalization, political freedom, financial development and institutional quality on environmental performance. The results of^30^ revealed that ethnic diversity, institutional quality and political freedom were significant influencers in mitigating CO_2_ emission while on the other hand energy consumption, GDP growth and financial development promotes environmental degradation. Thus, it was concluded that cohesion among ethnic groups, improvement in institutional quality, enhancing political freedom and participatory (inclusive) financial sector were [potential ways of enhancing pollution-free environment.

Wang et al.^31^ highlighted the relevance of institutional quality in enhancing environmental performance. It was reiterated that economy with weak institutions are characterized with financial misappropriation/embezzlement, breach of contract agreement, insecurity and general bad governance which are influencers of poor environmental quality. This is as a result of poor environment-related policies to reduce CO_2_ emission which such institutions may generate^32,33^. It is anticipated that an economy with solid and sound institutions are more likely to experience a prosperous economy, with relevant policies in place which aid mitigation of CO_2_ emissions^33–35^. Ulman and Bujancă^36^ also examined the role of economic indicators on environmental quality in Malaysia and found that environmental Kuznets curve model was not valid in Malaysia as a result of the U-shaped relationship between financial liberalization and ecological footprint (environmental degradation). Thus, it was suggested that Malaysia should promote energy efficiency and environmental well-being while increasing its GDP. Furthermore^37^, confirmed that economy with stable financial market possesses the potential to enhanced cleaner environment than countries with weak and unstable financial institutions. This is valid because stable financial institutions are viable drivers of foreign direct investment which invariably encourages EG.

Nigeria, the country with the largest black population and popularly called “The Giant of Africa” was presumed to experience increased economic growth in Africa, according to^38^. However, the economic downturn experience of 2016 had really impacted on the efforts of EG^37^. The recorded EG in the country was not without its negative impact especially on the quality of the environment. Between 1971 and 2010, the challenge of carbon emission (CO_2_), consumption of oil resources, deforestation and rising population had been on the increase with per capita GDP^37,39^ also identified the impacts of economic level and air pollution on public health at different levels and found, among others, that air pollution has negative correlation with public health and so, concluded that the higher the level of the economy, the less is the effect of EG in mitigating the effects of air pollution on public health. It was consequently suggested that sustainable economic growth should be pursued with reduction in air pollutants^23,40^. Ukaogo et al.^23^ identified income, financial development, energy consumption and trade as significant variables that explain environmental quality in Nigeria whereas, urbanization and income had unidirectional causality of environmental degradation. In view of this, it was suggested that cautions should be taken in committing funds to investment proposals to ensure low carbon emission.

This study was theoretically premised on the postulation of^41^ which was built from ecological point of view as postulated by^42^ and also, substantiated by^43^. According to^43^, the rise in the population (P), technological innovations (T) and financial strength (affluence) (A), encroachment into the natural environment (I) is inevitable. The duo, therefore, developed a model to elucidate on the relationship between the three variables as: I = P*T*A. In furtherance, Xie et al.^42^, in agreement with^43^, but from ecological point of view corroborated that as human population of a given space continues to rise, it is expected that pressure will be mounted on the available resources to improve living in that society. Such pressure is expected to exert consequences on the environment in terms of degradation of the environmental resources (sol, water, vegetation and air). Furthermore, in view of the pressure and the quest to make living in that society, some people will engage in agriculture (crop farming, animal husbandry and so on), commerce and other technical works. All these lead to damages on the natural environment. Dietz and Rosa’s model was later on modified by^41^ by including some other variables that also contribute to the quality of environment such as energy intensity, urbanization level, trade openness, industrial structure and transportation infrastructure. Generally, Xie et al. added to the model majorly carbon emission (green economy), population (household size), transportation infrastructure, affluence (economic power), technical progress (including waste management and waste sorting), energy utilization, level of urbanization (with living standard) and industry.

## Method of study

### Study area

**Figure 1:**
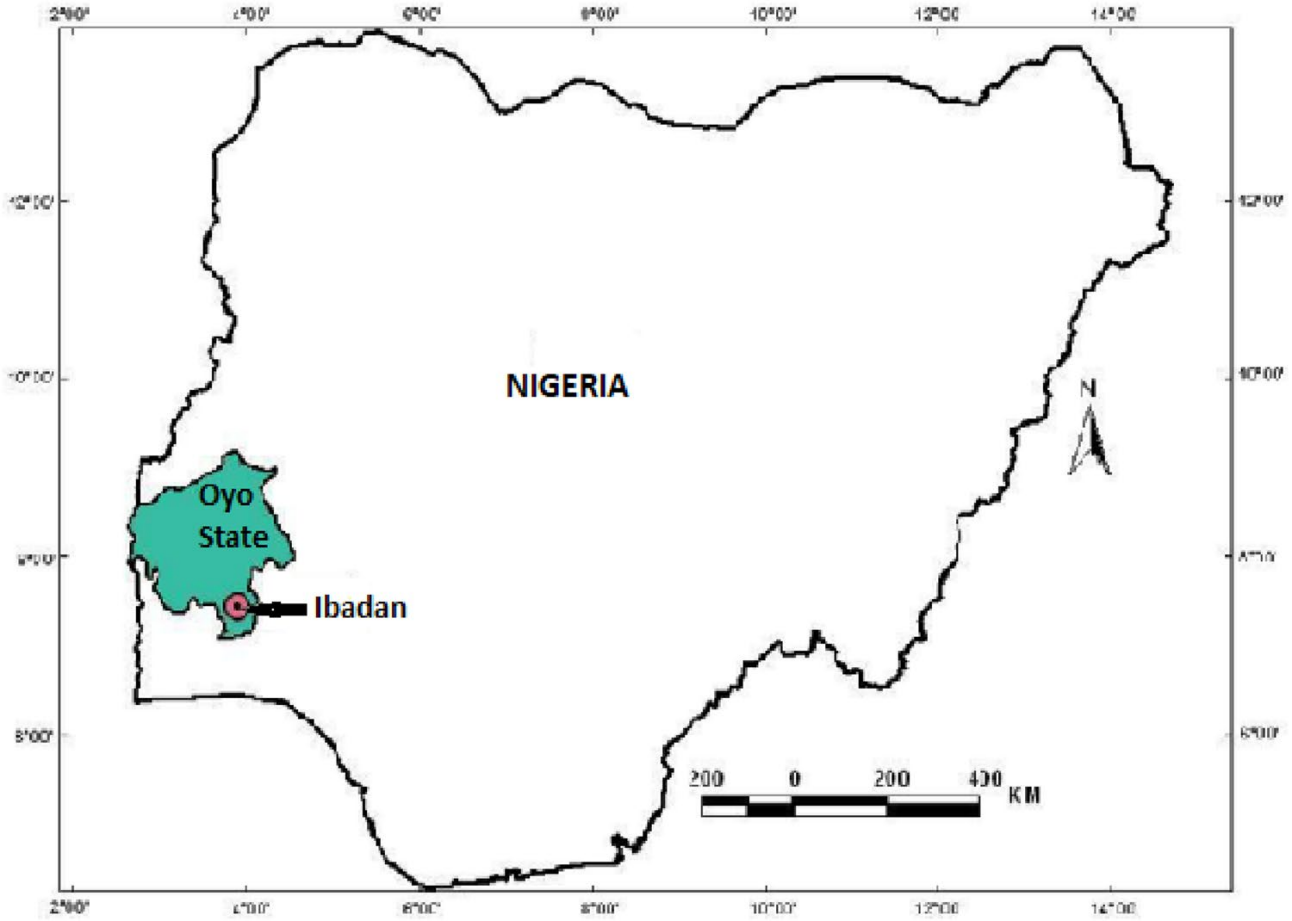
Map of Nigeria showing the location of Ibadan in Oyo State.

**Figure 2:**
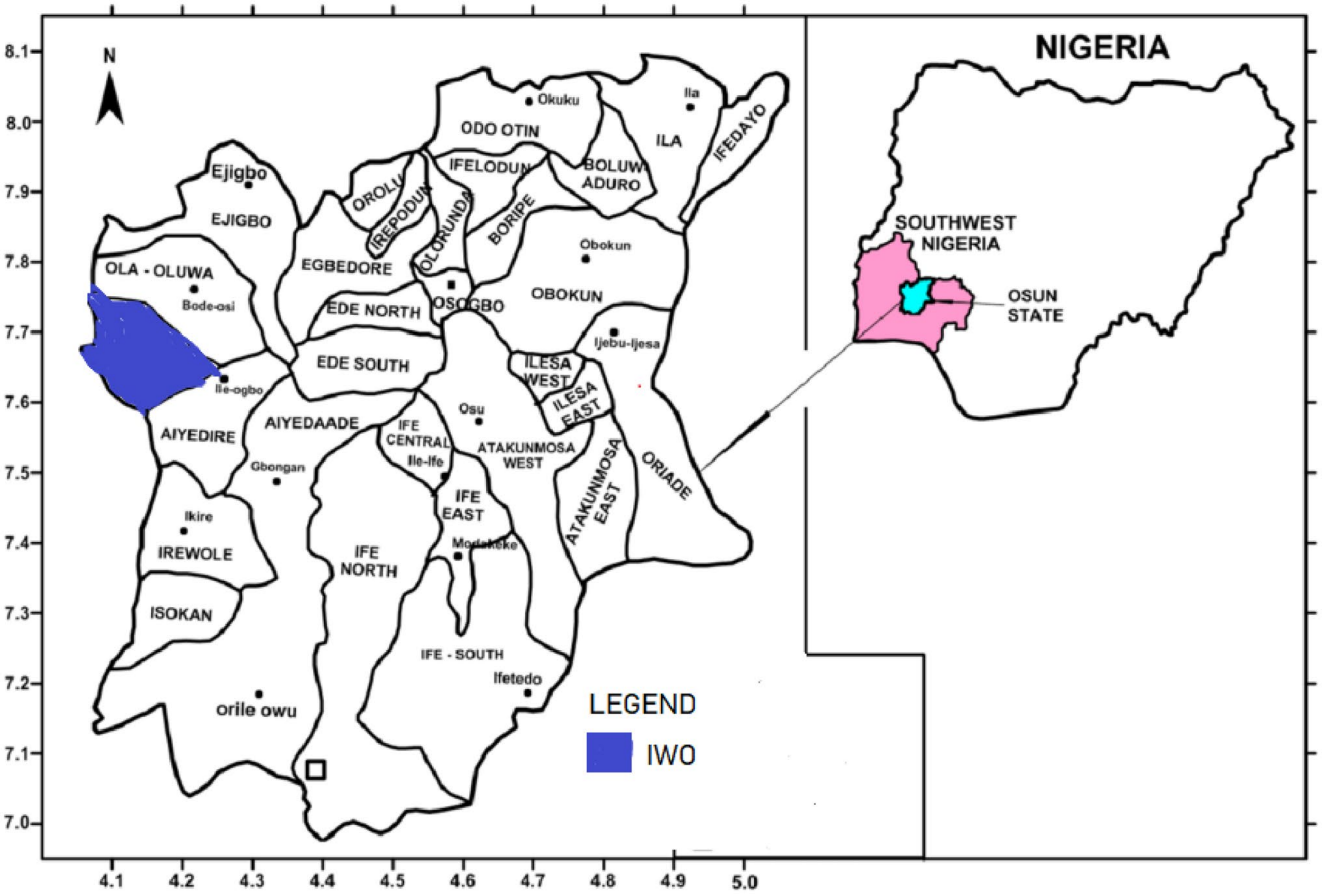
Map showing Iwo LGA in Osun State (Inset: Map of Nigeria showing Osun State).

Iwo and Ibadan (Figs. 1 and 2) are both located in the south western part of Nigeria but in different State. Iwo is one of the towns in Osun State and the headquarters of Iwo Local Government Area (LGA) located on the coordinates of 7° 63′ N and 4°18′ E. On the other hand, Ibadan is the capital city of Oyo State, Nigeria located on 7° 22′ N and 3° 56ʹ Both cities share boundaries towards the eastern part of Ibadan. Dietz and Rosa^44^ puts the population of Ibadan metropolis at 1, 887.100 for 2016. It is the zonal headquarters of Ibadan/Ibarapa zone of Oyo State with eleven LGAs out of which five are located within the municipality of the city. The five LGAs are Ibadan North, Ibadan North East, Ibadan North West*,* Ibadan South West and Ibadan South East covering an area of 3080 km^2^ making the city twelve times the area of Iwo. The growth of Ibadan can be traced to the early times when Ibadan became the seat of old Western Region in Nigeria Which comprised of the five (5) States in the South west namely Ondo, Ekiti, Ogun, Osun and Oyo State. In view of its status as the capital city of Oyo State, the city has attracted a lot of tertiary institutions and research institutes. Ibadan harbors named Bola Ige International Market (popularly called Gbagi Market), one of the biggest markets in Nigeria apart from other ones located within the nooks and cranes of the metropolis making the town a commercial city. Apart from being the location of educational institutions, Ibadan is also the location of many industries and many other historical infrastructures. For instance, the famous Cocoa House, Airport, University of Ibadan and so on are located in the heart of the city. These features of the metropolis have influenced the settlement of diverse of people from various of life and profession in the city. Ibadan has formed a nucleus for many other places thus making it a nodal city for both northward, eastward, westward and southward travelers. However, Ibadan has been noted for the problem of solid waste management which has been a serious challenge to the authorities in charge. The flood disaster of 1982, 1996 and 2003 which led to the destruction of life and properties worth millions of Naira have been attributed to the indiscriminate waste disposal in the metropolis.

On the other hand, Iwo, covering an area of 245 km^2^, is the headquarters of Iwo LGA. The population of the city according to^44^ by 2022 has been put at 263,500. Iwo is a typical agrarian economy depending on agriculture as its mainstay for livelihood. Iwo is well noted for its market popularly called Odo-Ori Market which often receives patronage of people from far and near including buyers and sellers from Ibadan among others. In term of industrial activities, Iwo is not endowed. Most of the medium scale industries include Gaari processing factories, oil palm mills. It is the sites or Bowen University, a privately-owned university owned by the Nigeria Baptist Convention, Westland University and other Islamic/Arabic schools.

### Data collection

The data used for this study was generated through the administration of questionnaire majorly. In some instances, some other relevant information was obtained through field observations. 165 questionnaires were completed and retrieved out of the 185 administered in Iwo survey station. The selection of the sample was based on the five quarters into which the town was divided namely; (1) Gidigbo, (2) Molete; (3) Isale Oba; (4) Oke Adan; and (5) Oke Oba^45^. Average of thirty-seven households were selected by simple randomization for the administration of the questionnaire. Two LGAs were investigated in Ibadan namely: Ibadan North East and Ibadan North LGAs out of the five (5) LGAs in the municipality for the purpose of generating data for Ibadan metropolis. The selection of the two LGAs was based on their accessibility for the researchers and the fund available for the work. Four hundred and eighty copies of the questionnaires were conducted in the two LGAs out of which 473 were completed and retrieved. Each of the LGA selected in Ibadan was gridded for the purpose of the survey of 258 households randomly selected in each. Household heads were selected for the survey where they are available, otherwise any available elder in each of the households sampled was selected in the survey.

### Data analysis

Descriptive and inferential statistical techniques were both used in the analysis of the data generated. The general characteristics of the respondents in each of the locations were presented in tabula form in percentages and averages. Factor analysis was used to determine and ordering the economic variables influencing pollution in the study areas using SPSS (version 16.0) software.

## Results and discussion

### Descriptive analysis

The general characteristics of the respondents in Iwo and Ibadan are presented in Table 1.

**Table 1:** Some attributes of the respondents involved in the survey.

S/no	Descriptive	Iwo	Ibadan
1	Gender		
	Male	85 (51.5%)	182 (38.5%)
	Female	80 (48.5%)	291 (61.5%)
2	Marital status		
	Married	129 (78.2%)	383 (81.0%)
	Single	30 (18.2%)	72 (15.2%)
	Widow	6 (3.6%)	18 (3.8%)
3	Level of education		
	Primary	45 (27.3%)	73`(15.4%)
	Secondary	79 (47.9%)	265 (56.0%)
	Tertiary	41 (24.9%)	135 (28.6%)
4	Monthly income		
	< N30,000.00	45 (27.3%)	112 (23.6%)
	30,000–60,000	61 (37.0%)	181 (38.3%)
	60,000–90,000	31 (18.8%)	102 (21.6%)
	91,000–120,000	15 (9.1%)	53 (11.2%)
	> 120,000	13 (7.8%)	25 (5.3%)
5	Household size		
	1–5	76 (46.1%)	231 (48.8%)
	6–10	54 (32.7%)	206 (43.6%)
	11–15	26 (15.8%)	24 (5.1%)
	16–20	5 (3.0%)	8 (1.7%)
	> 20	4 (2.4%)	4 (0.8%)

Table 1 showed that male respondents was 51.5% and 48.5% male and female respondents were respectively involved in the survey in Iwo, the respondents in Ibadan were 38.5 and 61.5 percent males and females in that order. This scenario could be attributed to the availability pattern in homes in study area. The study areas are part of Yorubaland in the southwestern Part of Nigeria, the tribe that holds the belief that women and children are meant to be at home to engage in home chores when compared with their male counterparts. This observation supported the findings of^46^ which asserted that the keeping of women at home is a necessity in other to protect them.

Also revealed in Table 1 is that more of the respondents in both study areas were of married status. The proportion was 78.2 and 81.0 percent respectively for Iwo and Ibadan while single men and women were respectively 31 and 37 percent in favour of Ibadan metropolis. The dominance of married respondents in the survey was on the premise that full details on the subject matter can be obtained than in singles’ homes. In fact, the single respondents were extremely accommodated when married were not available in any of the houses sampled. The preference of married respondents was to support the views of^2,19^.

Furthermore, Table 1 shows that respondents with secondary education levels formed the bulk of the people surveyed. While 47.9% were involved in Iwo, 56.0% were surveyed in Ibadan metropolis. This composition was deliberately for relative ease of interpretation and answering the questions made in view of the technicality involved in attending to the questions raised in the questionnaire. It was only where people with tertiary education were not available that the researcher engaged the respondents with less qualification. Thus, 18 and 10 percent of secondary level from Iwo and Ibadan respectively. However, the 27.3 and 15 percent of respondents with primary education in Iwo and Ibadan in that other were either assisted by the research assistant or by their educated children. This distribution was similar to the work of^47,48^.

The analysis further showed that 55.8 and 59.9 percent of the respondents in favour of Ibadan station earned between N30,000.00 and N90,000.00 while 27.3 and 23.6 percent earned less than N30,000.00, all on monthly basis in Iwo and Ibadan respectively. However, the data showed that 7.8 and 5.3 percent of the respondents earn N120,000.00 and above monthly in both Iwo and Ibadan stations respectively. The monthly income level is a reflection of the level of poverty on developing nations of the world and Nigeria, importantly as recorded by^49,50^.

The household size of the respondents as presented in Table 1 disclosed that 46.1 and 48.8 percent have maximum of 5 members in them while 37.7 and 43.6 percent have between 6 and 10 members, in that order for Iwo and Ibadan. Apart from this, 18.8 and 6.8 percent have 11 to 20 members in them in Iwo and Ibadan respectively while 2.4 and 0.8 percent have not less than 20 members in Iwo and Ibadan respectively. The distribution of household sizes is a reflection of the current trend in family procreation. Studies have shown that many homes, especially the elites and others that believe in small family size, in the contemporary times engage in monogamy rather the old practice of polygamy that encourages large family size in the both study areas. In another instance, many respondents with large family size probably are those that have their extended relations living under the same roof as a family. This fact is in support of the views of^51,52^.

### Identified economic attributes influencing pollution in Iwo and Ibadan metropolis

Six economic factors that have impacted on pollution in both Iwo and Ibadan metropolis were identified from the survey. The factors are (1) household income level (2) accommodation type and living standard (3) waste/noise management strategies (4) energy utilization (5) orthodox or green economy and (6) waste sorting needs.

1. *Household income level* Income level has a strong influence on household livelihood and standard of living, including mode of transportation, housing structures, safety and comfort among others
2. *Accommodation/Living standard* This factor indicates structure of the living apartments whether it is one-bedroom, two-bedroom, three-bedroom types, availability of functional plumbing and sewage system and the willingness to relocate to other communities
3. *Waste/Noise Management strategies* This parameter focused on recycling/reuse of wastes, use of alternative packaging materials, detoxification (a practice of avoiding the use of harmful chemicals in living areas, use of protective equipment such as boots, face-mask gloves and so on, cleanliness/sanitation culture and use of low noise turbo machines such as silencers and stereo de-amplifiers
4. *Energy utilization* This factor explains major sources of household energy in use in homes, type or mode of transportation;
5. *Inclination towards Green Economy* This parameter is about the availability of landscaping with trees and flowers, free availability of seedlings, frequency of waste removal on weekly basis (i.e. rural based job opportunities) and the ability to pay for refuse collections (measure of equity and welfare) outside the orthodox demand/supply curve; and
6. *Waste sorting* Household reasons for sorting wastes whether to make additional income or reduce the quantity and/or to convert degradable to composts.

### Some economic factors influencing pollution in the study areas using factor analysis

Factor analysis statistic was used to extract factors that strongly influence pollution out of the identified variables in either ascending or descending order. The factorability of the data was determined by KMO and Barttlets tests. The results of the tests revealed that the data is significant (*p* < 0.05) with KMO of 64.8% The summarized results of factor analyses for both study areas are presented in Tables 2 and 3.

**Table 2:** Summarized results of Analysis showing the Extracted Factors for Iwo.

s/n	Indices	Rotated component matrix	Rank (eigen value)	% of variance	Cumulative %
1	Waste/Noise management strategies	790	1.338	22.3	22.3
2	Accommodation/living standard	806	1.124	18.7	41.0
3	Inclination towards green economy	648	1.098	18.3	59.3

*Source* Culled from SPSS-generated results.

**Table 3:** Summarized results of Analysis showing the Extracted Factors for Ibadan.

s/n	Indices	Component	Total eigen value	% of Variance	Cumulative %
1	Accommodation/living standard	665	1.466	24.4	24.4
2	Inclination towards green economy	668	1.129	18.8	43.2
3	Waste/Noise management strategies	624	1.019	17.0	60.2

*Source* Culled from SPSS-generated results.

The results of the analysis revealed that three economic factors were extracted as having strong influence on the pollution in the study area as presented in Table 2. The three extracted factors for Iwo had a total explanation of 59.3% for the entire factors which exert influence on the pollution in Iwo. The factors were in this order: (1) Waste/noise management strategies which had the highest explanation for pollution level in Iwo of 22.3% which implies that waste recycling/reuse, avoidance of harmful chemicals among others are prominent in the town. The next important variable is the housing structure followed by orthodox or green economy with the least explanation for the pollution level in Iwo with 18.3%. The results corroborated the findings of^53^.

Similarly, three variables were extracted by factor analysis for Ibadan metropolis according to Table 3. The three factors in order of the magnitude of explanation given for the pollution level in the metropolis were (1) Accommodation/living standard (2) Orthodox or Green Economy and (3) Waste/Noise Management strategies. All the three variables gave a total of 60.2% explanation for the pollution in the metropolis. Accommodation and living standard which formed the most significant factor gave a proportion of 24.4%. This implies that the type of accommodation and or structure whether one-bedroom or two-bedroom and apartment and so on, availability of functioning plumbing and sewage system and the household’s willingness to relocate have great influence on the level of pollution in the metropolis. The importance of green technology was also mentioned by^54–57^ on ensuring environmental quality in their respective areas of studies.

From the ongoing, it is evident that the pollution matters in Iwo and Ibadan were both influenced by three but different variables. Even though two of the three factors were similar, their respective levels of significance in the order were not similar. While waste and noise management strategies was foremost in Iwo, it had the least explanation in Ibadan metropolis. Similarly, orthodox or green economy was least important in Iwo while it was a strong factor in Ibadan being the second on the order for Ibadan. Thus implying that Iwo and Ibadan should be treated separately from each other when seeking solutions to pollution-related matters. Rizam et al.^58^ and Ogunbode and Ifabiyi^59^ had similar results when it was discovered that each local government area should be treated individually when solving the water-related challenges.

## Conclusions and recommendation

Comparative analysis of the economic factors which influence pollution in Iwo and Ibadan metropolis has been carried out. The survey carried out showed that most of the respondents were of female gender, married status, tertiary education level and household size of between one and five. Six economic factors were identified and were subjected to factor analysis to determine the factors that were prominent in influencing pollution in the two communities. The results of the analyses showed that three different variables gave significant explanations for the pollution in Iwo and Ibadan. The significant variables extracted for Iwo that gave 59.3% of the explanation for the economic factors influencing pollution. These were given in that order: Waste/noise management strategies (22.5%), accommodation and living standard (18.7%) and orthodox or green economy (18.3%). The three important variables extracted for Ibadan explained 60.2% of the influence of all economic variables on pollution. These were given in the order of accommodation and living standard (24.4%), orthodox or green economy (18.8%) and waste/noise management strategies (17.0%). Though two of the variables namely accommodation and living standard, and orthodox or green economy were common to both study areas, their respective importance and ordering position varied. While waste and noise management was significantly in Iwo, the same variable had least explanation in Ibadan. Similarly, while inclination for orthodox or green economy had the least explanation for Iwo, It was more significant in Ibadan metropolis. It can, therefore, be concluded that economic factors that influence pollution in both Iwo and Ibadan metropolis differ and so efforts towards resolving pollution-related matters using economic indices should be treated separately one from the other.

## Data Availability

The datasets generated and/or analyzed during the current study are not publicly available the data used here were from a pull of data on which further investigations are still ongoing as at the time of submission but are available from the corresponding author on reasonable request.
